# Effective variant filtering and expected candidate variant yield in studies of rare human disease

**DOI:** 10.1038/s41525-021-00227-3

**Published:** 2021-07-15

**Authors:** Brent S. Pedersen, Joe M. Brown, Harriet Dashnow, Amelia D. Wallace, Matt Velinder, Martin Tristani-Firouzi, Joshua D. Schiffman, Tatiana Tvrdik, Rong Mao, D. Hunter Best, Pinar Bayrak-Toydemir, Aaron R. Quinlan

**Affiliations:** 1 Department of Human Genetics, University of Utah, Salt Lake City, UT USA; 2 Department of Pediatrics, University of Utah, Salt Lake City, UT USA; 3 Division of Pediatric Hematology/Oncology, Center for Children’s Cancer Research, Huntsman Cancer Institute, University of Utah, Salt Lake City, UT USA; 4 School of Medicine, Emory University, Atlanta, GA USA; 5 Department of Pathology, University of Utah, Salt Lake City, UT USA; 6 ARUP Institute for Clinical and Experimental Pathology, ARUP Laboratories, Salt Lake City, UT USA; 7 Utah Center for Genetic Discovery, University of Utah, Salt Lake City, UT USA; 8 Department of Biomedical Informatics, University of Utah, Salt Lake City, UT USA

**Keywords:** Sequence annotation, Genetics research

## Abstract

In studies of families with rare disease, it is common to screen for de novo mutations, as well as recessive or dominant variants that explain the phenotype. However, the filtering strategies and software used to prioritize high-confidence variants vary from study to study. In an effort to establish recommendations for rare disease research, we explore effective guidelines for variant (SNP and INDEL) filtering and report the expected number of candidates for de novo dominant, recessive, and autosomal dominant modes of inheritance. We derived these guidelines using two large family-based cohorts that underwent whole-genome sequencing, as well as two family cohorts with whole-exome sequencing. The filters are applied to common attributes, including genotype-quality, sequencing depth, allele balance, and population allele frequency. The resulting guidelines yield ~10 candidate SNP and INDEL variants per exome, and 18 per genome for recessive and de novo dominant modes of inheritance, with substantially more candidates for autosomal dominant inheritance. For family-based, whole-genome sequencing studies, this number includes an average of three de novo, ten compound heterozygous, one autosomal recessive, four X-linked variants, and roughly 100 candidate variants following autosomal dominant inheritance. The slivar software we developed to establish and rapidly apply these filters to VCF files is available at https://github.com/brentp/slivar under an MIT license, and includes documentation and recommendations for best practices for rare disease analysis.

## Introduction

Rare human diseases are often caused by de novo or inherited variants in a single protein-coding gene^1,2^. Isolating the small subset of causal variants from the numerous inconsequential variants in cohort exome and genome datasets remains an analytical bottleneck. The decreasing cost of sequencing has resulted in a dramatic increase in the number of groups analyzing sequence data from rare disease families. Because of alignment and variant calling artifacts^3^, careful filtering is required to extract an accurate set of causal variants. Each research group may choose custom strategies, use ad hoc software, or one of many tools designed to facilitate the filtering, including seqr (https://seqr.broadinstitute.org/), GEMINI^4^, and genmod (https://github.com/moonso/genmod). This leads to innumerable possible outcomes when analyzing the same cohort.

Even within a single tool, different parameter values can affect the number of candidate variants, which in turn, can impact variant prioritization and the ability to reach a genetic diagnosis. A previous study^5^ examined filtering strategies on variant call files (VCFs) but did not provide a set of parameters that can be widely applied across cohorts or the expected number of candidate rare-disease variants expected. Here, we introduce a minimal, but effective set of filtering parameters and report the resulting number of candidate variants for each mode of inheritance. We sought to provide a set of variant filtering parameters that would reduce variability across studies and provide a common baseline for research across different tools and research groups. We have avoided several common filtering strategies that are either cohort-specific, or potentially too strict; for example, although prioritizing predicted loss-of-function (pLoF) variants^6,7^ can reduce the search space, many pathogenic variants in ClinVar^8^ are not pLoF, so most analyses must include a broader set of variants. These filters were found to have nearly identical performance in two whole-genome cohorts and also in two whole-exome cohorts. The resulting set of recommended, data-derived filters can be used in any tool as a standard practice for variant filtering. The strategies we describe establish a baseline expectation for variant counts per trio for each inheritance mode.

Throughout, we note that counts of autosomal dominant candidates differ by orders of magnitude from the number of candidates from de novo dominant and recessive modes of inheritance. Therefore, researchers exploring autosomal dominant candidates will require additional filtering or prioritization, though here, we limit our explorations to simple, strict filtering and report the resulting counts.

## Results

### Establishing allele-balance and genotype-quality thresholds for exome studies

**Fig. 1:**
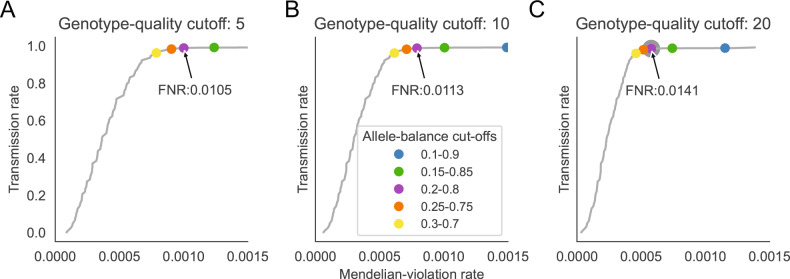
Evaluation of the impact of allele-balance and genotype-quality cutoffs on Mendelian violation rates for trio exomes. We measured the number of Mendelian violations (*x*-axis) and transmissions (*y*-axis) as we varied allele balance within each plot. The genotype-quality cutoff applied is increased from 5 (**A**) to 10 (**B**) to 20 (**C**) for each plot. The line in each plot is drawn by varying the allele-balance cutoff and counting the number of variants that are predicted to be transmitted or apparent Mendelian violations. Dots in each plot indicate the exact rates at a given threshold. The chosen cutoff, marked with an asterisk, required a genotype quality 20 and an allele balance between 0.2 and 0.8. The false negative rate (FNR) for the allele-balance cutoff of 0.2–0.8 (in purple) is annotated for each genotype-quality cutoff.

Using a cohort of 149 mother, father, and child “trios” that had been exome sequenced (see “Methods”), we labeled variants as potential Mendelian violations when the parents were predicted by GATK^9^ to be homozygous for the reference allele, yet the child was predicted to be heterozygous. Because we expect between zero and two true de novo variants per exome trio^2^, Mendelian violations in excess of this expectation are predicted to be false positives. Alleles that were heterozygous in the child and in only one parent were considered to be transmitted from the parent to the child, and treated as true positives. We varied allele balance (AB; i.e., the ratio of reads aligned at a variant locus that support the alternate allele) cutoffs before declaring a variant to be either a Mendelian violation or transmitted variant (Fig. 1). For transmitted variants, we used the minimum AB between the parent and the child as the value that was filtered in creating the curve. The flat portion of the curve indicates parameter changes that remove likely spurious violations while retaining transmitted variants.

These results established a genotype-quality (GQ) cutoff of 20 or higher and an AB between 0.2 and 0.8 (the purple point in each figure) as a rational trade-off between precision and sensitivity, as it removes many of the Mendelian violations (false positives) while retaining most (98.6%) transmitted variants (true positives). In fact, this is likely a conservative threshold, as even the more stringent threshold of 0.3–0.7 has a very high transmission rate, and an estimated false negative rate of ~1.41%. In our testing, this range performed well for both exome and genome data (see below). We performed the same analysis on an independent cohort of 36 whole-exome trios from a study of congenital heart disease at the University of Utah. We verified that the curves were similar and that resulting thresholds were similarly effective (Supplementary Fig. 2). Specifically, we note that the performance curves are similar, that the thresholds are on a nearly identical location in each curve, and that the chosen thresholds strike a similar balance of sensitivity and specificity. For consistency with genome cohorts, we also applied a depth threshold to exome calls (Supplementary Figs. 1 and 2), which further reduced the violation rate. While this cutoff is a reasonable default, it is simple for users to adjust these AB cutoffs if, for example, more stringent calling is desired. Not surprisingly, this recommendation is similar to thresholds used elsewhere^10^. Nonetheless, to our knowledge, it is the first data-driven derivation of filtering cutoffs.

### Evaluation of filters on the number of predicted de novo variants in exome studies

**Fig. 2:**
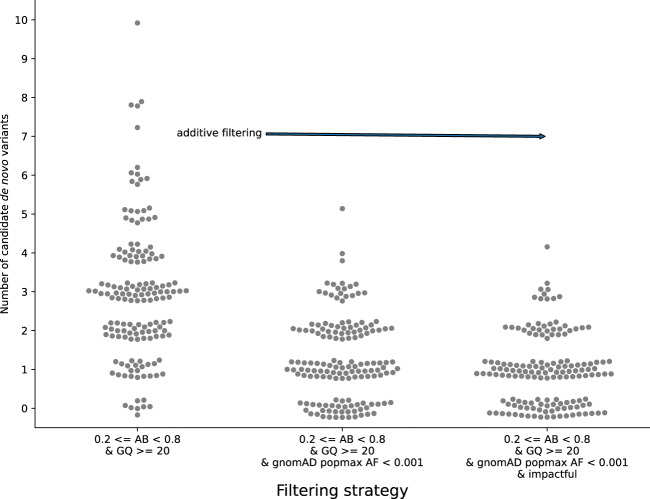
The effect of combined filters on the number of predicted de novo mutations in exome studies. The number of candidate de novo variants for each of 149 exome trios. In each column, a point represents the number of de novo mutations per trio. Moving right along the plot, each column adds filters to the column that precedes it. The first column uses only the sample information derived above, where AB is allele balance (alternate reads/(alternate reads + reference reads) and GQ is genotype quality. The second column adds filters on gnomAD allele frequency (AF); this reduces the average number of candidates. The third column further requires that the variant is “impactful,” according to slivar.

In addition to filtering variants based on GQ and AB, for rare disease research it is also important to require a candidate de novo variant to be rare or absent in population databases such as gnomAD^11^. Given the rarity and severity of the phenotype in rare disease studies, it is common to examine variants predicted to have high (e.g., variants that introduce a stop codon or alter the coding frame) or moderate (e.g., amino acid altering variants) impact of the resulting protein. Such variants are henceforth referred to as “impactful.” Combining these additional requirements reduces the number of candidate de novo mutations from a mode of 3 to a mode of 1 per exome trio (Fig. 2). These filters can be used across inheritance modes, except that the minimum population (gnomAD) allele frequency (AF) for recessive modes should be relaxed because selection on recessive alleles only arises when frequencies are high enough that a (pathogenic) allele from each parent can be transmitted to a child. While it is common to filter de novo mutations by allele count in the population to filter out pipeline-specific artifacts^10^, we chose not to utilize this as it would require a larger cohort and diminish the generality of our variant filtering guidelines.

### Candidate variants predicted across multiple inheritance modes for exome studies

**Fig. 3:**
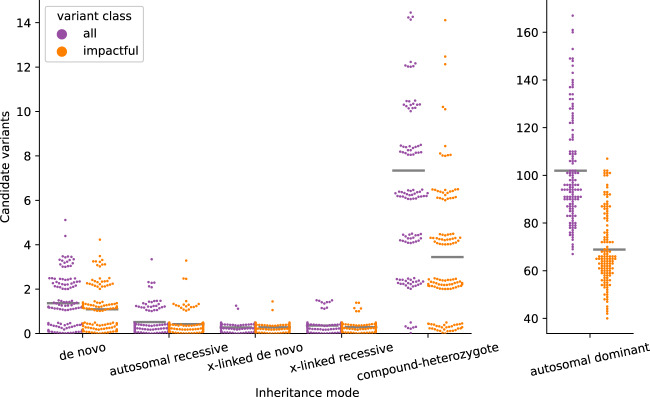
The number of candidate variants that follow different inheritance modes per exome. The number of candidate variants for 149 exome trios are separated by inheritance mode and colored by variant class. Variants deemed impactful by slivar using annotations from VEP, snpEff, and bcftools. Counts for autosomal dominant variants are shown in a separate plot due to the much larger numbers. Each point represents the number of candidate variants for a single family (*y*-axis) passing the inheritance mode (*x*-axis), genotype-quality, population allele-frequency, and allele-balance filters. Gray bars indicate the mean number for each class and inheritance mode. Points are jittered slightly to allow viewing more samples simultaneously.

Integrating these thresholds, we evaluated the number of candidate variants identified for a typical exome trio under de novo, autosomal recessive, compound heterozygote, X-linked recessive, X-linked de novo, and autosomal dominant inheritance models (Fig. 3). Under de novo and autosomal dominant models of inheritance, we required candidate variants to have an AF < 0.001 in each of the eight gnomAD populations (e.g., African, Latino, East Asian, etc.). For all others, we required a frequency <0.01, and for autosomal dominant, we also required that the number of homozygous alternate alleles in gnomAD to be <10.

We emphasize that for exomes, filtering for “impactful” variants does not dramatically reduce the number of variants per sample, and is therefore not required for rare disease analysis (for example, compare the middle and right columns in Fig. 2). Therefore, in exome studies, we recommend the use of impactful as an annotation, rather than as a strict filter such that all variants are reported, while only a subset is marked as impactful. A key insight from this analysis is that, except for dominant inheritance modes, we can expect around ten candidate variants when applying data-driven population AF, GQ, and AB filters to a typical exome.

### Evaluation of filters on the number of predicted de novo variants in whole-genome studies

We repeated similar analyses for a cohort of 94 trios that underwent whole-genome sequencing (WGS) to ~30X coverage, as part of the Rare Genomes Project (“Methods”). In order to explore additional options for WGS variant filtering, we considered calls from DeepVariant^12^ that were joint-genotyped by GLNexus^13^ and compared filtering strategies with GATK^9^ calls. Using only AB and GQ filters, GATK reports more ostensibly transmitted variants than DeepVariant and GLnexus (hereafter referred to simply as “DeepVariant”) at the expense of a higher number of Mendelian violations (Supplementary Figs. 3 vs. 4, top row). Supplementary Figs. 3–10 depict performance curves for Mendelian violations vs. transmissions for various parameter combinations for two cohorts for both GATK and DeepVariant. This represents a large volume of parameter-space but we note that in every plot, the general shape of each curve is similar and the location of our chosen cutoff (the purple dot) is effective but conservative.

With our means of choosing transmitted variants as true positives, it is possible that the additional transmitted variants in GATK relative to DeepVariant are merely false positives shared between parent and child. Yun et al. found that while GATK makes more calls than DeepVariant, DeepVariant actually has a higher recall, indicating that many of the extra GATK calls are actually potential false positives^14^.

Based on the analysis in Supplementary Figs. 3–10, we decided to use the same AB and GQ thresholds for genomes as for exomes. We found that, for each candidate variant, requiring at least ten aligned sequences in all members of the trio and excluding low-complexity regions^3,9^ retained most transmitted variants and removed a large percentage of Mendelian violations reported when no sequencing depth requirement was enforced across cohorts and tools (Supplementary Figs. 7–10). Mendelian violation rates increase when variants in low-complexity regions are included (Supplementary Figs. 3–6 vs. 7–10). Projects with higher coverage or different accuracy requirements may raise or lower the depth threshold, but we considered this to be an acceptable trade-off. We validated that these thresholds were effective in an independent cohort of whole genomes with diverse ancestry revealed by PCA analysis with Peddy^15^ (see Supplementary Fig. 11). In addition, we performed an additional evaluation using the Genome-in-a-Bottle’s (GiaB) set of high-quality variant calls^16^ to show that each of our genotype, allele-balance, and depth thresholds removed false-positive calls and retained true-positive calls in an independent evaluation set (Supplementary Fig. 12). Specifically, the chosen GQ cutoff alone would remove >80% of false positives and retain >99% of true positives in GiaB. The depth and AB cutoffs would each remove about 20% of false positives and retain >99% of true positives.

**Fig. 4:**
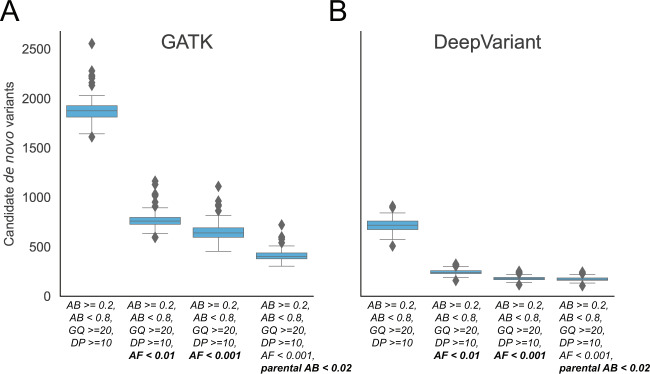
Candidate autosomal de novo variants per genome identified by GATK and DeepVariant outside of low-complexity regions. A cohort of 94 WGS trios from the Rare Genomes Project were screened for candidate de novo mutations using GATK (**A**) and DeepVariant (**B**). Variants lying in low-complexity regions were excluded. The leftmost boxplot within each subplot requires a depth ≥10, an allele balance between 0.2 and 0.8 along with a genotype quality (GQ) ≥20. Lines within the boxplot are determined from the quartiles of the data. The next box requires that the allele frequency in gnomAD is <0.01. The third box lowers the allele-frequency cutoff in gnomAD of <0.001. The final box excludes candidate de novo variants where the allele balance (of the homozygous call) in the parent is ≥2%. Supplementary Fig. 13 presents the analogous plots when including low-complexity regions.

We note that even with depth and parental AB filtering, we are left with a median of 3127 and 660 candidate de novo variants per trio from GATK and DeepVariant, even after excluding variants in low-complexity regions (see Supplementary Fig. 13). We know from previous studies^10,17,18^ that an average of ~70 de novo SNV and INDEL mutations should be observed genome-wide; therefore, the vast majority of these predicted mutations are false positives. Filtering on depth (DP), AB, genotype quality, AF, and parent allele-balance reduced the number of putative de novo variants from both GATK and DeepVariant calls (Fig. 4). Note that while this initially seems a daunting rate of error in these variant callers, we are enriching for errors by nature of the search for rare, de novo mutations. In fact, with each of the four million expected variant sites in an individual, this number represents an extremely low false-positive rate for variant detection (~0.07% for the median of 3127), even if the false discovery rate for de novo mutations is relatively high prior to filtering. Because low-complexity regions are such a common source of false positives^3^, we excluded variants in those regions from consideration in Fig. 4. We further required that homozygous reference calls in the parents had an AB of <0.02. This substantially reduced the number of candidate de novo variants. However, there were still 407 putative de novo variants from GATK and 172 from DeepVariant outside of low-complexity regions. While DeepVariant reports fewer variants, it is still more than twice the expected number^10^ and would require additional filters to reach a reasonable number. However, when limiting to impactful variants, the number drops to an average of 1.5 and 3.3 candidates for DeepVariant and GATK, respectively—a number small enough such that each candidate variant could be scrutinized. With a less stringent population AF filter of <0.01 in gnomAD, there are 1.7 and 7.4 candidate variants from DeepVariant and GATK, respectively. These filters are lenient enough to be generalizable. However, specific projects may have additional filters, but we argue that these averages are low enough to be reasonable guidelines when considering “impactful” variants as is typical for studies of rare disease.

**Fig. 5:**
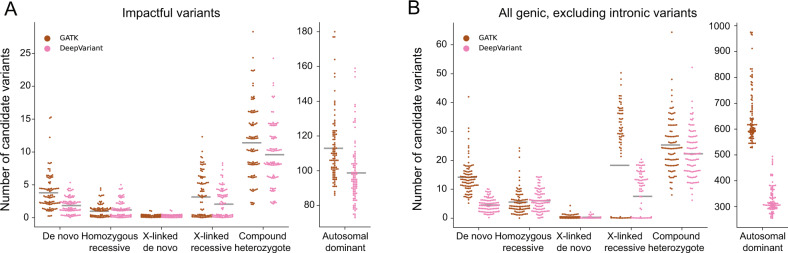
The number of candidate variants that follow different inheritance modes per genome using two different variant callers. **A** Only “impactful” variants as determined by slivar using annotations from VEP, snpEff, or bcftools are shown. **B** The set of variants is extended to include synonymous, UTR, and conserved intron regions (but not all intronic). Counts for autosomal dominant variants are shown in a separate plot due to the much larger numbers. Each dot represents the number of candidate variants (*y*-axis) passing the inheritance mode (*x*-axis), genotype-quality, population allele-frequency, and allele-balance filters for a single family. Gray bars indicate the mean number for each class and inheritance mode. We show Fig. 5A for the sarcoma replication cohort in Supplementary Fig. 14.

With the general filtering strategies outlined above, we examined the number of candidate “impactful” variants discovered in each WGS trio under each mode of inheritance (Fig. 5). Although DeepVariant reports about half as many putative false-positive calls as GATK (see Fig. 4), after filtering with gnomAD AF (below 0.001 for de novo and dominant and below 0.01 for recessive modes of inheritance), the difference between the two callers is substantially reduced (Fig. 5A). This effect is similar to what was observed for exomes, but with generally higher variant counts due to the additional genes and coding regions covered in whole-genome data. Note that for genomes, expanding the search to include synonymous and UTR variants can more than double the number of compound heterozygote, x-linked recessive, and autosomal dominant candidates (Fig. 5B). Across all modes of inheritance, the total increase when allowing less impactful variants is large enough that additional prioritization strategies might be needed. However, including all intronic variants further increases the number of candidates by several fold. These findings are replicated in an independent Ewing sarcoma cohort in Supplementary Fig. 14 and Supplementary Table 1.

Though we show that we retain nearly all ostensibly “true” variants with our filters, we also assessed whether our filters excluded candidate causal variants reported by the RGP project. In short, we recovered all expected SNP and indel variants. For samples we evaluated, we recovered 21 of the 24 single-nucleotide or insertion-deletion variants reported as causal by the RGP Project. Variants that were discovered by the RGP team but not reported in this analysis were limited to those where one side of a compound heterozygote was a CNV call (CNVs were not evaluated in the present study) or to nonstandard inheritance patterns (see Supplementary Discussion for additional details).

### Recommended practices and resulting candidate yield

We have shown that simple cutoffs on GQ, AB, depth, impact, and gnomAD AF are sufficient to drastically reduce the number of candidate variants in a typical family, while retaining the vast majority of transmitted variants (Table 1). We have avoided using cohort-specific attributes such as allele count to improve the generality of our findings. In order to evaluate variant calling accuracy, we use Mendelian violations as putative false positives, and heterozygous variants transmitted from one parent (and homozygous reference in the other parent) as true positives. While there are limitations to the assumptions underlying this approach (for example that the parent and child could share a spurious heterozygous call by chance or that somel Mendelian violations are not false positives), the shape of the ROC curves in Fig. 1 and Supplementary Figs. 1–10 demonstrate that certain parameter ranges dramatically reduce the number of Mendelian violations without reducing the count of transmitted variants. This indicates that parameter values beyond these ranges (e.g., low GQ, low depth, extreme ABs) are enriched for false positives.

**Table 1:** Recommended filtering parameters.

Description	Filter
High genotype quality for all samples in a family	GQ ≥ 20
Allele balance between 0.2 and 0.8 for heterozygous samples	0.2 ≤ AB ≤ 0.8
Allele balance <0.02 homozygous samples	AB < 0.02
Depth of at least 10 for all samples in a family in whole-genome data	DP ≥ 10 (whole-genome only)
Low allele-frequency (or absent) in population allele-frequency databases	population_AF < 0.01 (recessive inheritance modes)
	population_AF < 0.001 (dominant inheritance modes)

These simple strategies can reduce candidate “impactful” variants across most inheritance modes (excluding autosomal dominant) to a manageable number. While we expect these to vary by cohort, the numbers are similar across our whole-genome and whole-exome cohorts. For our whole-genome cohort, we find a mean of 13.1 (1.4 de novo, 0.8 autosomal recessive, 9.2 compound heterozygotes, 1.7 x-linked recessive, and 0.02 x-linked de novo) and 18.3 (3.5 de novo, 0.7 autosomal recessive, 11.2 compound heterozygotes, 2.9 x-linked recessive, and 0.0 x-linked de novo), for DeepVariant and GATK, respectively (Table 2). Over half of those variants (9.2 on average for DeepVariant and 11.2 for GATK) are pairs of variants for a compound heterozygote, so the number of candidate genes is even lower. These numbers of candidate variants replicated extremely well in a separate cohort of 49 ancestrally diverse family trios where the proband had Ewing’s Sarcoma (See Supplementary Table 1). While this number of variants is small enough that it is possible to manually inspect each variant in small cohorts where the mode of inheritance is not autosomal dominant, large cohorts will likely benefit from variant prioritization tools that are outside of the scope of this paper and of the slivar tool we developed for this study. When considering autosomal dominant modes of inheritance, the number of candidate variants will be high enough (average of ~100 candidates) that larger families, gene lists for limiting search space, or variant prioritization methods will be needed to discover causal variants reliably. In addition, once candidate impactful variants are ruled out, methods for limiting and prioritizing candidate variants from introns and noncoding regions will be essential. Likewise, when expanding beyond impactful variants in WGS studies, the count of candidate variants rises quickly; for example, from an average of 3.5 to an average of 434.7 GATK candidate *de novo* variants (Table 2). When not limiting to impactful variants, other prioritization methods or prior knowledge such as genes or regions of interest would be required to limit candidates to a reasonable number. We find that whole-genome samples have a much higher proportion of impactful variants with an impact of splice_region as compared to exome (Supplementary Fig. 15); this, combined with more complete coverage explains the higher counts in whole-genome studies compared to exome^19^.

**Table 2:** Mean number of candidate variants for each inheritance mode for a cohort of 149 whole-exome trios and and a cohort of 94 whole-genome trios.

Inheritance mode	Exome GATK	Genome GATK	Genome DeepVariant	Exome GATK impactful	Genome GATK (impactful)	Genome DeepVariant (impactful)	Genome GATK (genic except intronic)
De novo	1.4	434.7	178.7	1.1	3.5	1.4	13.9
Autosomal recessive	0.5	185.0	320.7	0.4	0.7	0.8	5.3
Compound heterozygote	7.3	21.9	21.9	3.4	11.2	9.2	25.0
X-linked recessive	0.3	572.7	401.8	0.3	2.9	1.7	18.0
X-linked de novo	0.3	5.0	2.5	0.3	0.0	0.0	0.2
Autosomal dominant	102.0	10,874.1	11704.6	68.9	112.9	99.0	653.2

## Discussion

We have derived minimal, yet effective filtering parameters for rare disease research and demonstrated their efficacy in rare disease cohorts having undergone exome or genome sequencing. Although the recommendations we make here may be intuitive, we argue that it is important to define clear, reproducible, and defensible recommendations as a starting point for rare disease analyses. We have also reported an expected number of candidate variants for each inheritance mode for exome and genome.

For clarity, our recommendations come as a single number (for example, GQ at or above 20). However, our analyses demonstrate that overall performance is not affected by the exact choice. For example, one could choose a GQ of 15 and an AB range of 0.3–0.7 and achieve a similar enrichment of true positive variants. Most likely, cohorts with lower sequencing depth would need to lower the depth cutoff of 10 (at the likely expense of additional false positives). This will be an avenue for future research.

While the exact choice of what is impactful will affect the results, the set of impacts used for this study would retain 98.83% of ClinVar^8^ pathogenic variants. This cutoff is easily changed by users of slivar by editing the default impact order; the ordering used in this study is included in Supplementary Table 2. Note that the impactful cutoff is currently quite inclusive by default, including for example “splice_region” in addition to the “splice_acceptor” and “splice_donor” sites. It is possible to expand the search to include UTR and synonymous variants. Including these categories can yield more than three times as many total candidate variants as compared to the impactful set. However, in some scenarios, this is still a small enough set to evaluate, but will require previous knowledge or variant prioritization methods to limit variants to high-quality candidates. Such knowledge will be even more necessary when searching introns and the noncoding genome. By providing clear data-driven variant filtering guidelines to filter variants, we provide reproducible strategies to limit the number of variants that may need to undergo more intensive investigations. These results memorialize, for example, the fact that the number of autosomal dominant variants is large enough that additional filtering strategies such as limiting to gene lists or utilizing variant pathogenicity scores would be required.

In summary, we have derived a set of best practices for variant filtering in studies of rare human disease, and report the expected number of candidate variants across different modes of inheritance. In addition, we have developed slivar, a new tool for rapidly applying these filters and extracting variants that meet each inheritance mode. We facilitate application of these recommendations with the continued development of slivar software.

## Methods

We have complied with all relevant ethical regulations. We have obtained informed consent from all relevant participants. The clinical exome data obtained from ARUP Laboratories for the main exomes for this study was approved for de-identified research use by the Institutional Review Board of the University of Utah.

### Software implementation

Slivar is implemented as a command-line tool. It is built on hts-nim^20^, which is a nim language wrapper for htslib. While slivar uses hts-nim for reading and writing VCFs, it also embeds the duktape javascript engine (https://duktape.org) to enable user-defined expressions on each variant. slivar expects a pedigree file that indicates the phenotype status and the family relationships; from this, it infers each possible trio and family. Then, for each variant, it fills a javascript object for variant and for INFO and for each trio, it consecutively aliases the samples with the labels “kid,” “mom,” and “dad” and then applies the user expression. For example, a minimal expression to call a de novo variant in the kid would look like:

kid.het && mom.hom_ref && dad.hom_ref && \ kid.GQ > 10 && mom.GQ > 10 && dad.GQ > 10 && \ variant.FILTER = = “PASS” && INFO.gnomad_popmax_af < 0.001

This expression requires that: the child is heterozygous while the parents are homozygous; each sample has a GQ > 10, and that the alternate allele for variant is observed at <1 in 1000 frequency in gnomAD. Slivar automatically discovers each trio from a pedigree file and applies this expression to each trio, for each variant. For any variant that meets these criteria for a given family, slivar appends the sample name of the kid to the variants INFO field in the VCF file. The attributes available for each sample (kid, mom, dad) and for INFO and variant are enumerated in Table 1. Expressions like the one above can be encapsulated in javascript functions, put in a separate file and then called, for example as:

denovo(kid, mom, dad)

This allows the distribution of a set of best-practices functions for de novo, compound heterozygotes, recessives, X-linked, and other modes of inheritance.

The trio mode described above is a special case of a more general framework within slivar for families. A family in slivar is any set of samples with the same family identifier in a pedigree file; this can be a single sample, or a large, multigenerational pedigree. This framework enables a more flexible set of expressions that handle most use cases, for example, finding a segregating, dominant variant in a single sample, a trio, a larger nuclear family, or a multigenerational pedigree is handled by a single javascript function.

Another mode, that we call “groups,” is an additional way to handle sets of samples that do not meet a normal family structure, for example, a cohort of cancer patients with a “normal” and “tumor1,” “tumor2,” “tumor3” to indicate three tumor time-points. These groups can be defined in slivar via a tab-delimited file where the header indicates the labels and each row indicates the sample IDs those labels are applied to. For example, a cohort with ten quartets would have a header line with four columns (in this case “kid,” “mom,” “dad,” “sib” might be appropriate) and ten rows, each with four sample IDs. The user expression utilizing the labels “mom,” “dad,” “kid,” “sib” would be applied to each of the ten quartets for each variant.

A user can specify multiple group, family, and trio expressions, each with a label that is added to the INFO field for each passing expression. For example, if an expression labeled as “denovo” is evaluated as true in a trio, then denovo = $kid_sample_id would be added to the INFO for that variant. Multiple samples (trios) passing the same expression are joined by commas.

### Compound heterozygote analysis

Because slivar expressions operate successively on each variant, there is a separate subtool to find compound heterozygotes. It expects gene annotations as added by snpEff^21^, VEP^22^, or bcftools csq^23^ in order to group variants by gene. It then uses the pedigree structure to phase variants to ensure that the two alleles in the compound heterozygote are on different haplotypes. In addition, it supports variant pairs where one side of the compound is a de novo. As with the standard slivar mode, this adds an annotation to the INFO field indicating the sample and the variant pair that are part of the compound heterozygote.

### Impactful variants

Annotations added by VEP, snpEff, or bcftools are automatically parsed by slivar and evaluated for “impactful-ness.” In slivar, we have collected all consequence annotations (missense, synonymous, frameshift, etc.), given them a severity order, and split them into “impactful” and not. This ordering is somewhat arbitrary, as for example a case could be made that stop_loss annotation indicates a more severe impact than frameshift or vice versa, but we have based it on the ordering indicated for variant-effect predictor (https://m.ensembl.org/info/genome/variation/prediction/predicted_data.html). In addition, the exact ordering is less important as a user will be interested in a variant that is stop_loss or frameshift should it appear. We have chosen the cutoff for impactful to be quite lenient, and the ordering and cutoff are both customizable by a simple text file. slivar will automatically detect and parse these annotations if they appear in the VCF, iterate over each consequence and add an “impactful” flag if any consequence is above the cutoff. This can be used independently of the family-based analyses to annotate variants of interest. If multiple annotations are found, for example from both VEP and snpEff, slivar will use the highest impact across tools to determine impact. This simple flag allows the user to find variants of interest without checking for an exact consequence of “missense,” “stop_gained,” etc.

For evaluating an expanded definition of impactful, we also included variants annotated as “synonymous,” “gene,” “coding_sequence,” “mature_miRNA,” “5_prime_UTR_premature_start_codon_gain_variant,” “5_prime_UTR,” “3_prime_UTR,” “initiator_codon,” “miRNA,” “non_coding_transcript_exon,” “non_coding_exon,” “nc_transcript,” “exon_region,” and “conserved_intron,” with UTR and intronic annotations capturing nearly all of the additional variants. We used this order of variants: https://github.com/brentp/slivar/blob/a79595f1dc5b6e7bb348f5c9b938e7866b70ab99/src/slivarpkg/default-order.txt.

### Annotation with population AF

As we demonstrate, annotating with population AF is critical for rare disease research. As the size of population AF resources such as gnomAD^11^, TopMED^24^, and dbSNP^25^ increase, distributing these resources and leveraging them for variant annotation is ever more time and resource-consuming. For example, the whole-genomes summary file from gnomAD v3 is 235 GiB. In order to facilitate annotation with massive population AF databases, we developed, as a part of slivar, a reduced format that can be easily distributed and used for rapid annotation and concurrent filtering. While there are solutions such as vcfanno^26^ for annotating VCFs, the size of these files (the gnomAD v3 VCF is ~235 GB and the V2 exomes file is ~59 GB) make them substantial requirements and slow to parse. To alleviate this, we developed a custom, reduced annotation format that can encode the variant position, reference allele, alternate allele, and a boolean indicating a non-PASS FILTER in a single 64-bit integer. This is similar to VariantKey (https://www.biorxiv.org/content/10.1101/473744v3), except that VariantKey stores the chromosome, but not the FILTER. slivar assumes that variant files will be sorted by chromosome, so we can store variants from each chromosome separately instead of including the chromosome in the encoded value. slivar stores variants with a REF + ALT allele longer than 13 bases in a separate text file as these can not be encoded into a 64-bit integer. Since the percent of variants of that size is low, the size of the text file is small and searching in the text file is only done when the query variant is also large. Whenever a long variant is found, a sentinel value with an empty reference and alternate allele are added to the encoded array indicating the presence of a long variant. Additional fields containing the values of interest, for example the AF and the number of hom-alt samples are stored in separate arrays. All arrays and chromosomes are written to a single compressed zip file. This allows us to distribute gnomAD version 3 AFs and positions for the whole-genome cohort in a 4-GB file (compared to the original 235-GiB compressed VCF). In order to annotate a query VCF, each query variant (position, reference, alternate) is encoded to the 64-bit value and a binary search is used to find that variant. If it is found, the index of that variant in the array is used to extract the values, which are then added to the INFO field of the query variant. If a sentinel value is found, slivar searches the (sorted) array of long alleles and returns those. This setup allows us to annotate at >20K variants per second. We call this format and annotation method “gnotation.” slivar performs this gnotation step before the user expressions are applied so that the expressions can utilize population AFs. This annotation can be performed independently of the family expressions as a way to quickly annotate with AFs.

Slivar includes a subtool to create these gnotation files, but we provide downloads for gnomAD for version GRCh37 and for hg38 that contain “gnomad_popmax_af,” and “gnomad_nhomalt” that are the union of exome and whole genomes. In addition, we provide a TOPMed gnotation file for hg38.

### Best-practices workflow for rare disease

In order to develop the best practices for rare disease, we utilize a cohort of 149 rare disease exome trios and a WGS cohort of 94 trios aligned to hg38. These families all have unaffected parents, making them more likely to follow recessive or de novo inheritance, however, we also evaluated autosomal dominant strategies by artificially setting the mother to “affected.” This gives an idea of the number of variants left after filtering in an analysis under each of these disease models. We also replicated the analyses in independent whole genome and exome cohorts. The additional whole-genome cohort contained 49 trios where the proband had a sarcoma phenotype and the exome cohort was 36 trios with a coronary heart disease phenotype.

We use the number of putative de novo and transmitted variants discovered in each trio to inform viable filtering strategies. We know, for example, that there should be between 0 and 2 de novo variants in the exome. Due to alignment issues, base-calling errors, and variant calling errors, there will be many more than this without additional filtering. We can evaluate the specificity of different filters by looking at the number of putative de novo variants; likewise, we^27^ can evaluate the sensitivity by counting the number of transmitted variants—that is, the number of heterozygous variants in one (and only one) parent that are transmitted to the child. We chose to require transmitted variants to appear in the child and in either, but not both parents, so that we would penalize a caller that over-called heterozygotes. If we allowed a true positive to be any variant that appeared in both parents and the child, it would be more likely that we included variants that were false positives in all three samples.

We primarily rely on filtering strategies that utilize allele balance, genotype quality, and genotype that will be common to most VCFs, rather than on custom fields like fisher-strand bias or mapping-quality rank-sum, which are optional annotations from GATK. Although depth is generally used, this can vary between cohorts and does not offer improvement over allele balance and genotype quality in exomes, therefore we do not utilize it as a default. But it can be beneficial in filtering whole-genome samples. In addition, for the whole-genome cohort we use Deep Variant^12^ and evaluate slivar filters effective for that tool.

For this study, we used nextflow^27^ to run slivar with and without low-complexity regions for each cohort and tools. This workflow used is available at the following URL: https://github.com/brentp/slivar/blob/cf9328054a84ac6c1c2400925df604fe3e8170b2/paper/slivar.nf.

### Reporting summary

Further information on research design is available in the Nature Research Reporting Summary linked to this article.

## Supplementary information

Supplementary Information

Reporting Summary

## Data Availability

RGP sequence data are available via: https://raregenomes.org/data-sharing. Ewing Sarcoma data are in dbGap Study Accession: phs001228.v1.p1. CHD data are in dbGap Study Accession: dbGaP phs000744. The clinical exome data obtained from ARUP Laboratories for the main exomes for this study were approved for de-identified research use by the Institutional Review Board of the University of Utah. However, ARUP legal counsel has determined that the clinical testing consent form signed by these patients does not allow for the full sharing of raw data to any publicly available database. The genome-in-a-bottle data are available from: ftp://ftp-trace.ncbi.nlm.nih.gov/giab/ftp/release/AshkenazimTrio/HG002_NA24385_son/NISTv4.1/GRCh37/.

## Code availability

Code used for the analyses in the paper is available here: https://github.com/brentp/slivar/tree/master/paper. The slivar software is available at https://github.com/brentp/slivar.
