# Supplementary material for: Effective variant filtering and expected candidate variant yield in studies of rare human disease
Source: NPJ Genom Med. 2021 Jul 15;6:60. doi: 10.1038/s41525-021-00227-3 (PMC8282602; doi:10.1038/s41525-021-00227-3)
Supplement: Supplementary file 1 — Supplementary Information [file 41525_2021_227_MOESM1_ESM.pdf]

Supplementary Figures

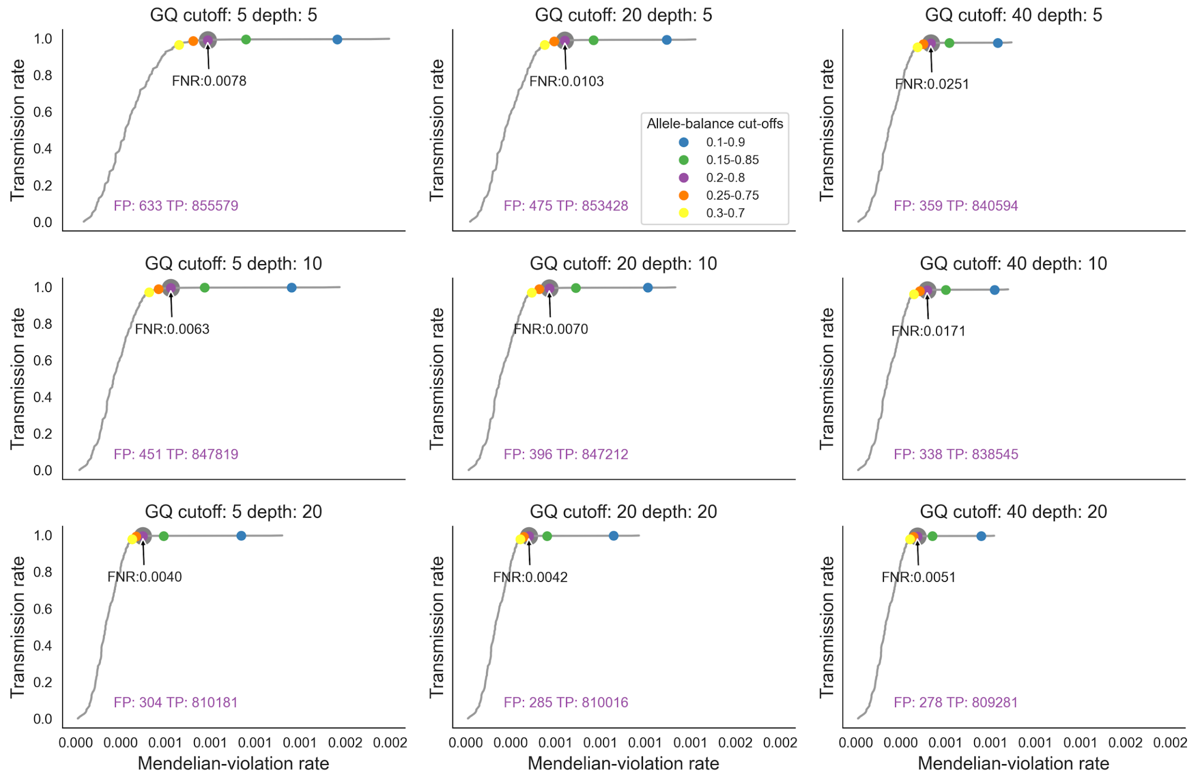

Supplementary Figure 1. Exome cohort 1 Curves GATK. Evaluation of the impact of allele balance, genotype quality, and depth cutoffs on Mendelian violation rates for trio exomes. We measured the number of Mendelian violations (x-axis) and transmissions (y-axis) as we varied allele balance within each plot. The genotype quality cutoff applied is increased from 5 to 20 to 40 across each row and the depth requirement is increased from 5 to 10 to 20 down each column. The line in each plot is drawn by varying the allele balance cutoff and counting the number of variants that are predicted to be transmitted or apparent Mendelian violations. Dots in each plot indicate the exact rates at a given threshold. The chosen allele balance cutoff is shown as the purple dot in each chart. We additionally required a genotype quality 20 and depth of 10 which is the center chart in this figure. The false negative rate (FNR) for the chosen allele-balance cutoff is annotated for each genotype quality cutoff.

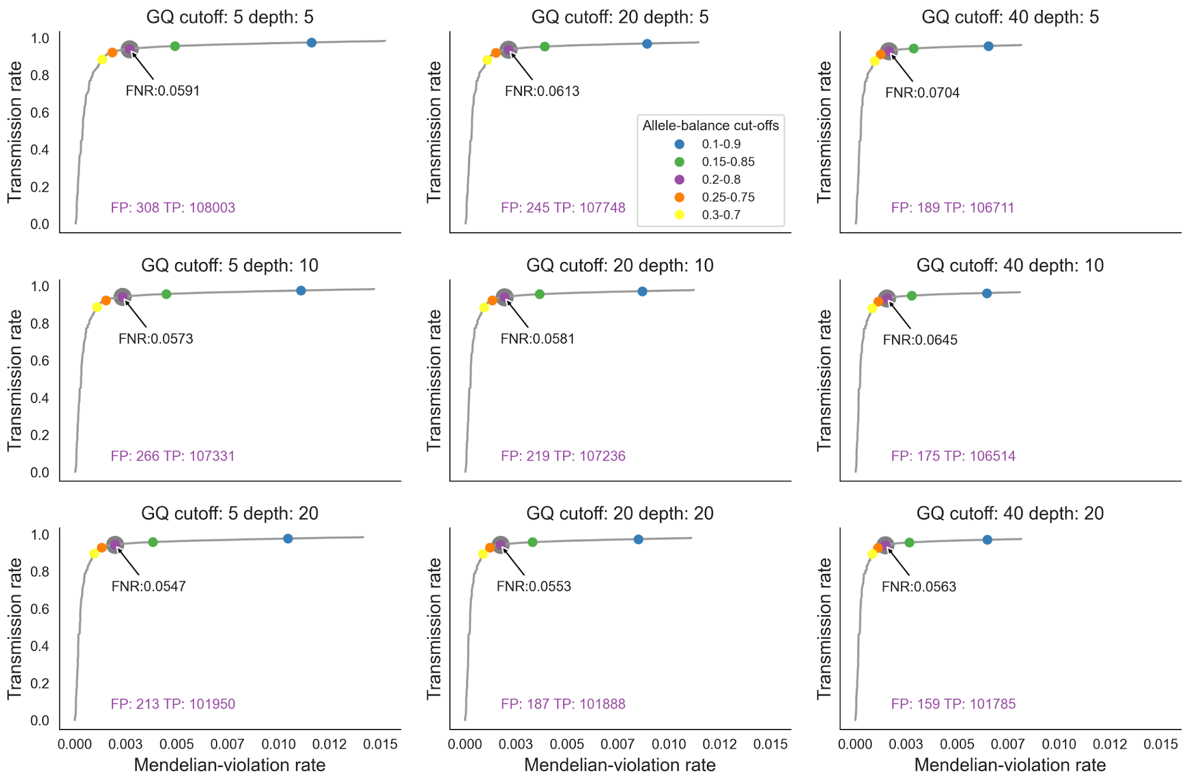

Supplementary Figure 2. Exome cohort 2 Curves GATK. See Supplemental Figure 1 for description of plot methods.

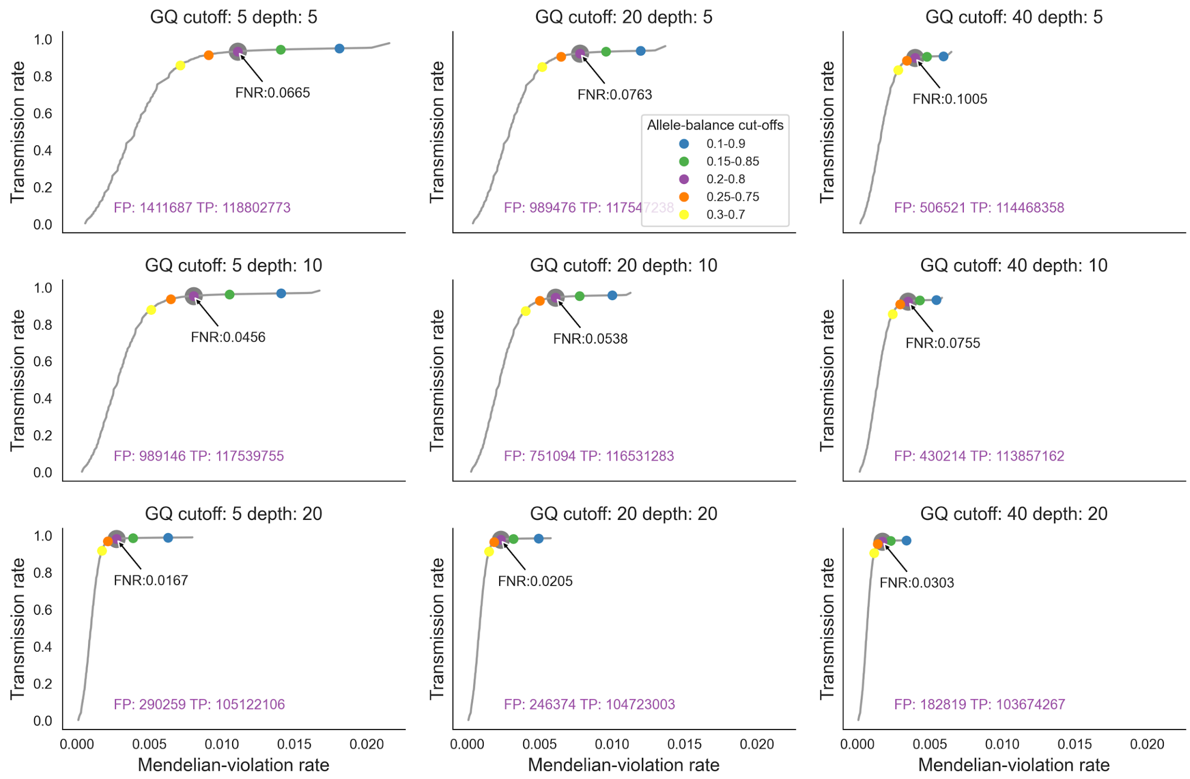

Supplementary Figure 3. Genome cohort 1 Curves GATK. See Supplemental Figure 1 for description of plot methods.

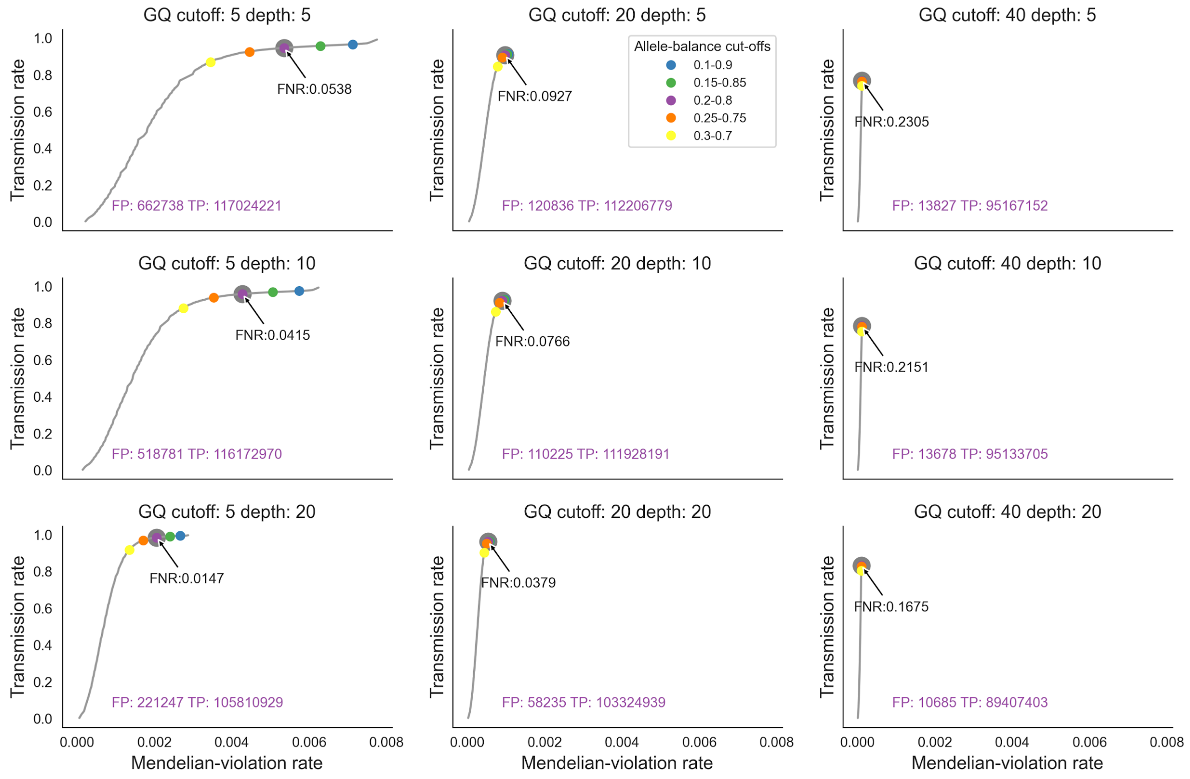

Supplementary Figure 4. Genome cohort 1 Curves DeepVariant. See Supplemental Figure 1 for description of plot methods.

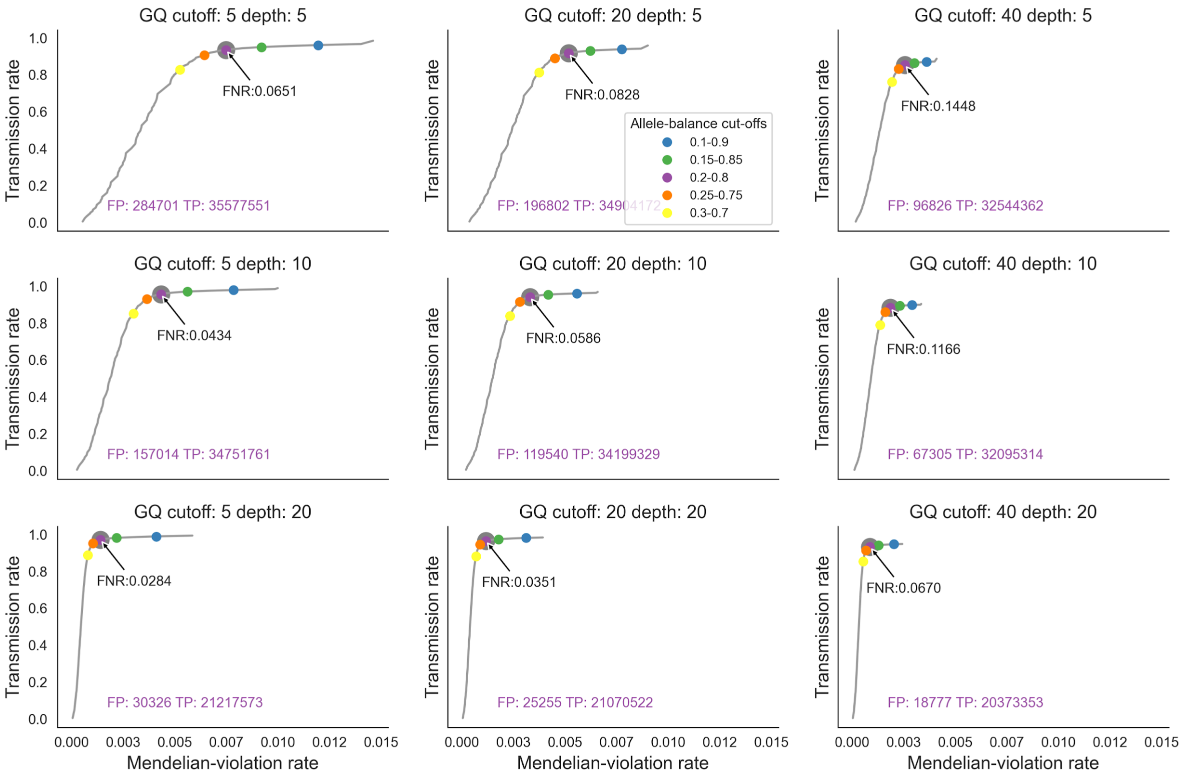

Supplementary Figure 5. Genome cohort 2 Curves GATK. See Supplemental Figure 1 for description of plot methods.

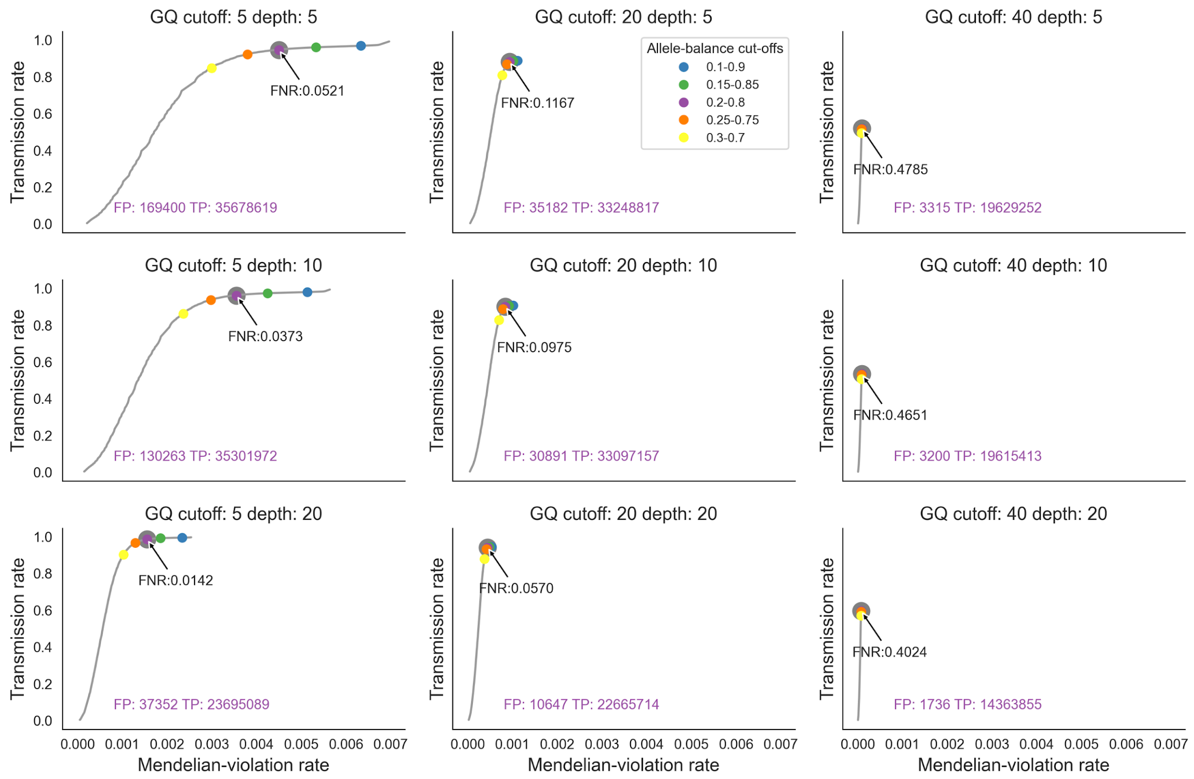

Supplementary Figure 6. Genome cohort 2 Curves DeepVariant. See Supplemental Figure 1 for description of plot methods.

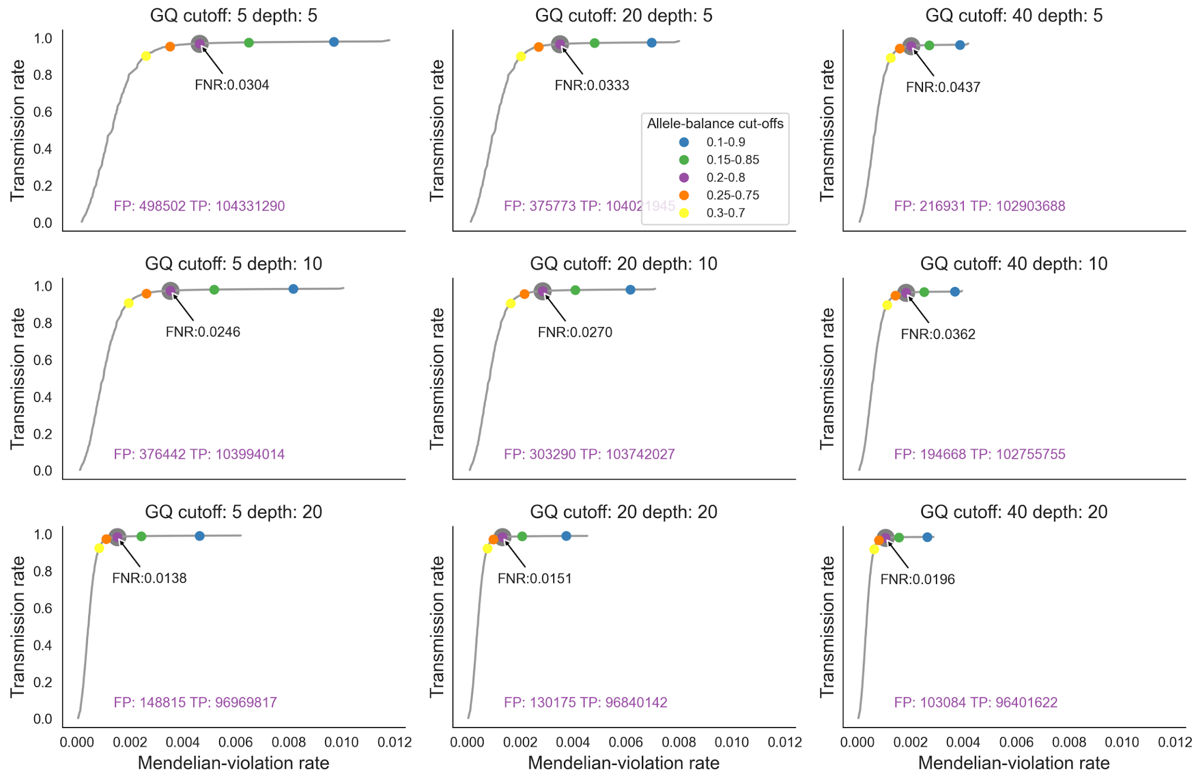

Supplementary Figure 7. Genome cohort 1 Curves GATK Excluding LCR. See Supplemental Figure 1 for description of plot methods. Additionally, this plot includes only variants outside of low-complexity regions.

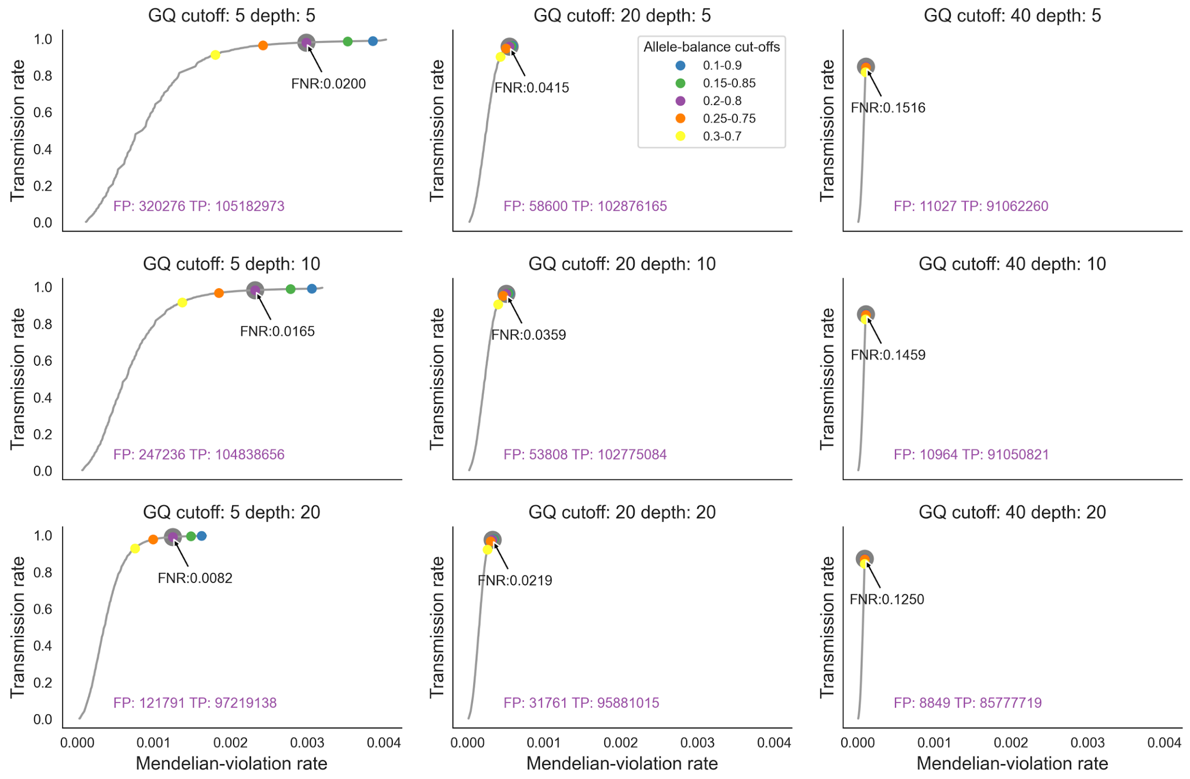

Supplementary Figure 8. Genome cohort 1 Curves DeepVariant Excluding LCR. See Supplemental Figure 1 for description of plot methods. Additionally, this plot includes only variants outside of low-complexity regions.

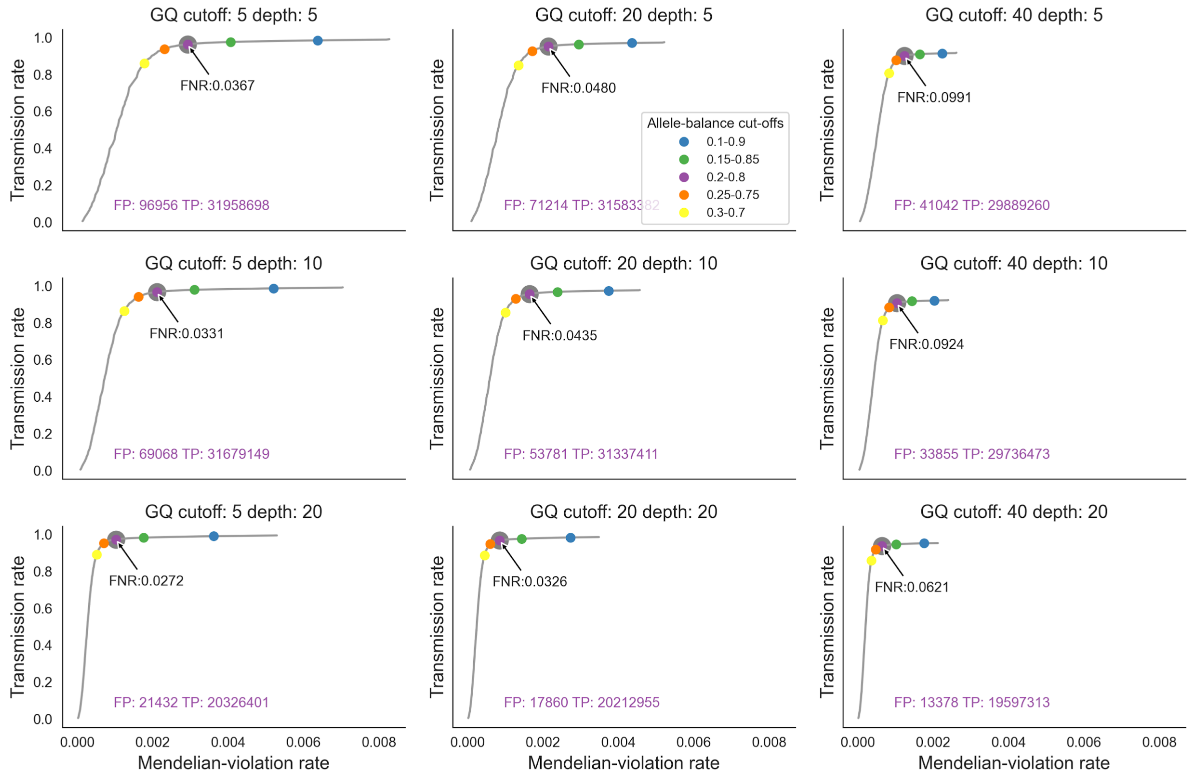

Supplementary Figure 9. Genome cohort 2 Curves GATK Excluding LCR. See Supplemental Figure 1 for description of plot methods. Additionally, this plot includes only variants outside of low-complexity regions.

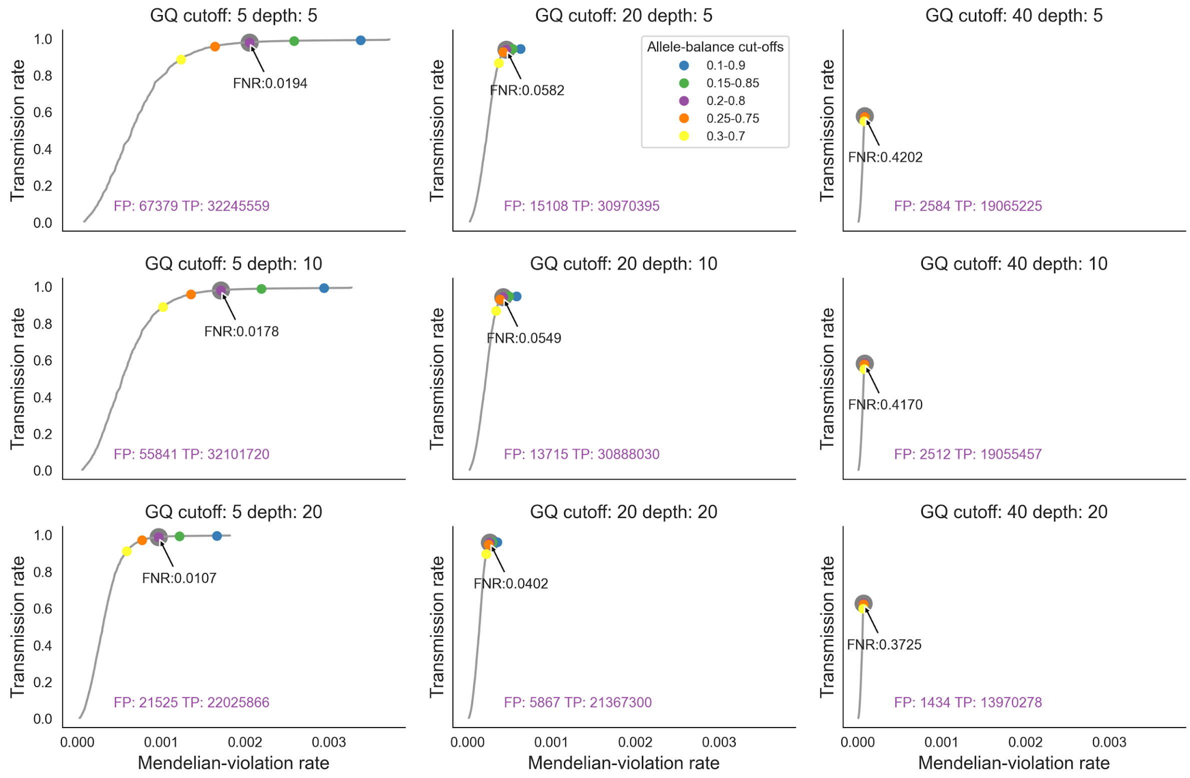

Supplementary Figure 10. Genome cohort 2 Curves DeepVariant Excluding LCR. See Supplemental Figure 1 for description of plot methods. Additionally, this plot includes only variants outside of low-complexity regions.

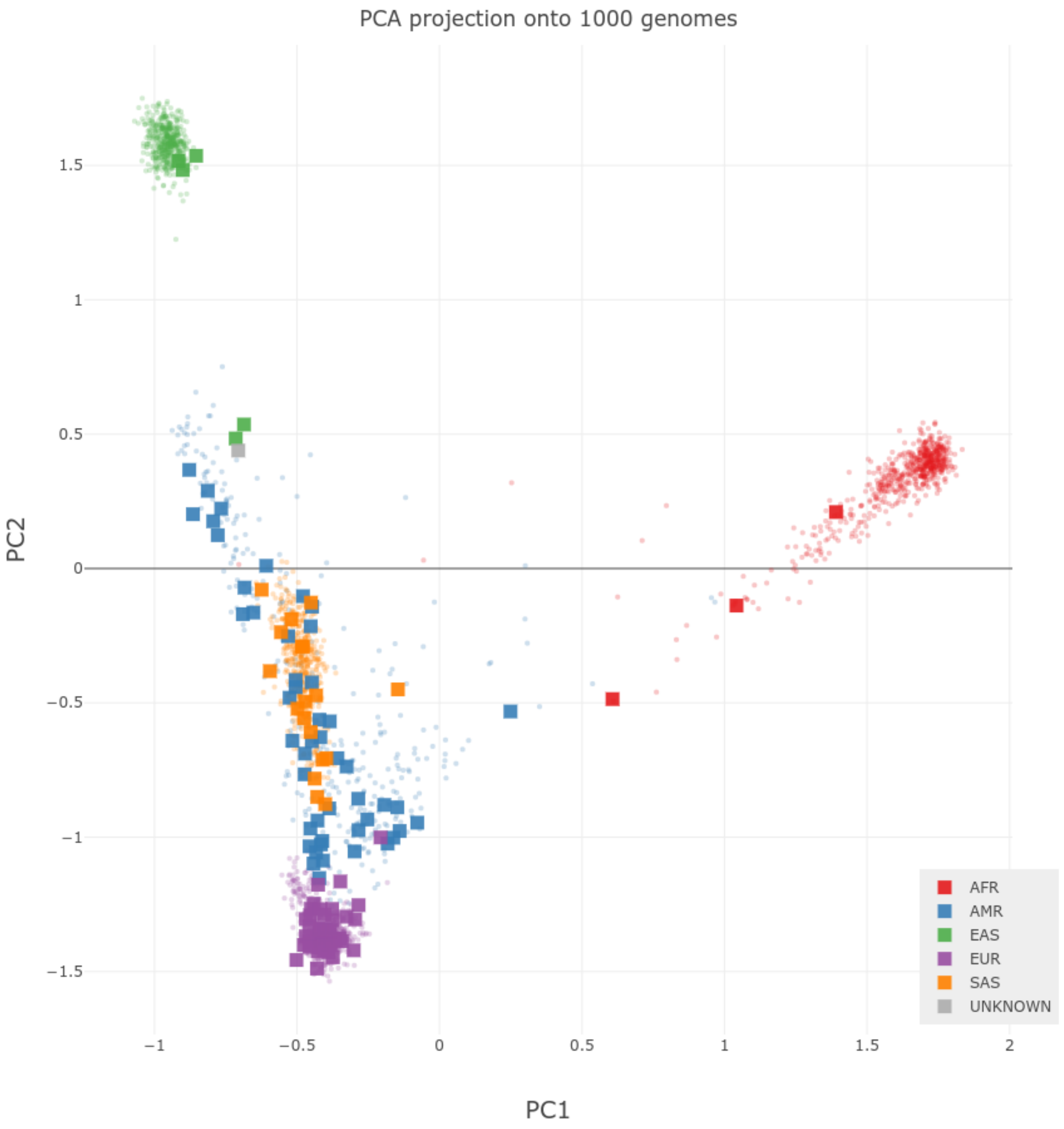

Supplementary Figure 11. Peddy Ancestry PCA for Sarcoma whole genome cohort. Each point is a sample colored by ancestry inferred by peddy using thousand genomes samples as training data. This plot shows that we have several non-european (AMR, SAS, AFR, and EAS) ancestries represented in the Ewing's Sarcoma cohort.

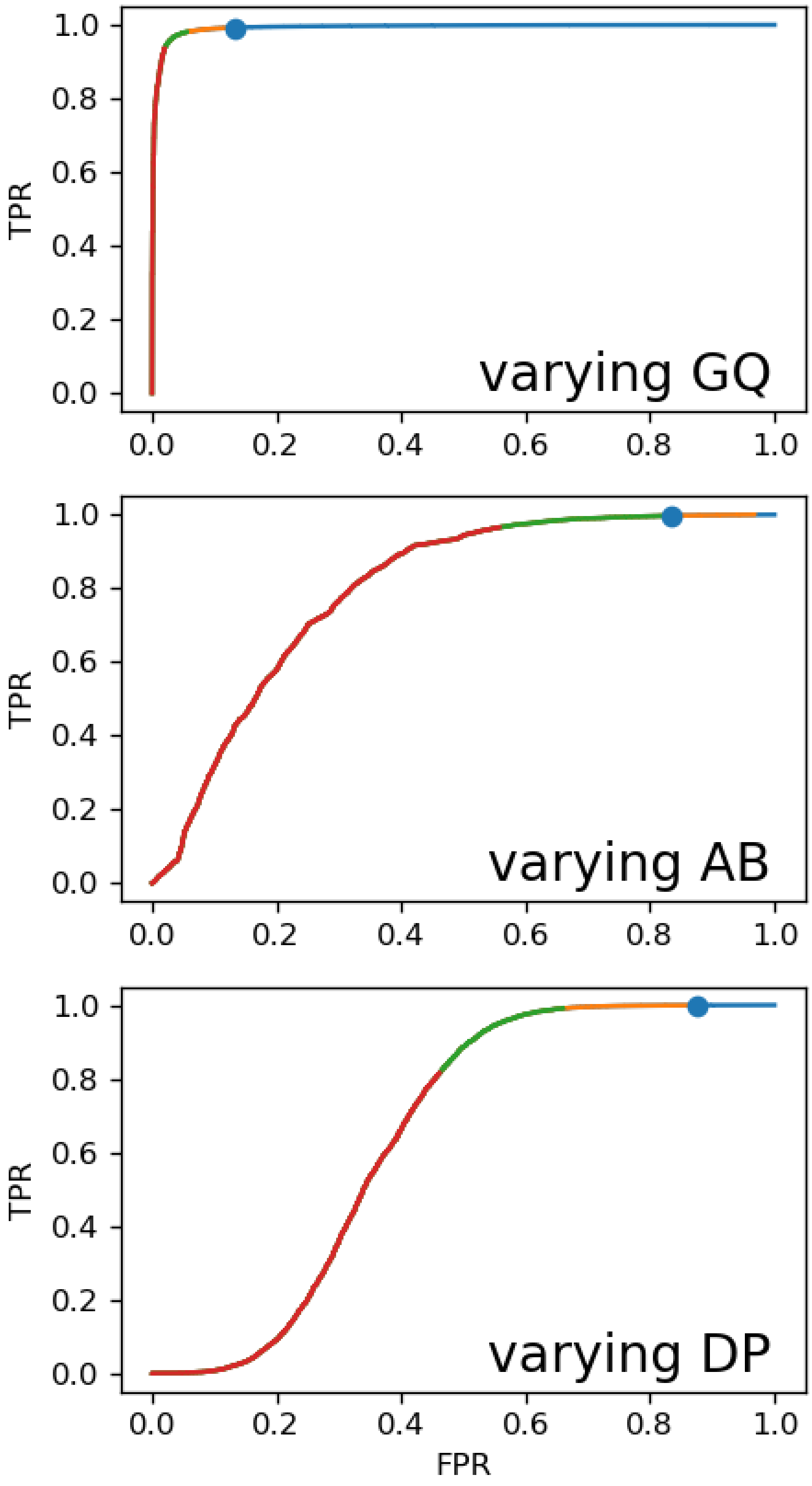

Supplementary Figure 12. Evaluation of GQ, AB, and DP cutoffs on Genome-in-a-bottle truth set. The blue dot in each subplot shows the cutoff we have chosen (GQ >= 20, 0.2 <= AB <= 0.8, DP >= 10). In all plots, the cutoff removes false-positives and retains true positives).

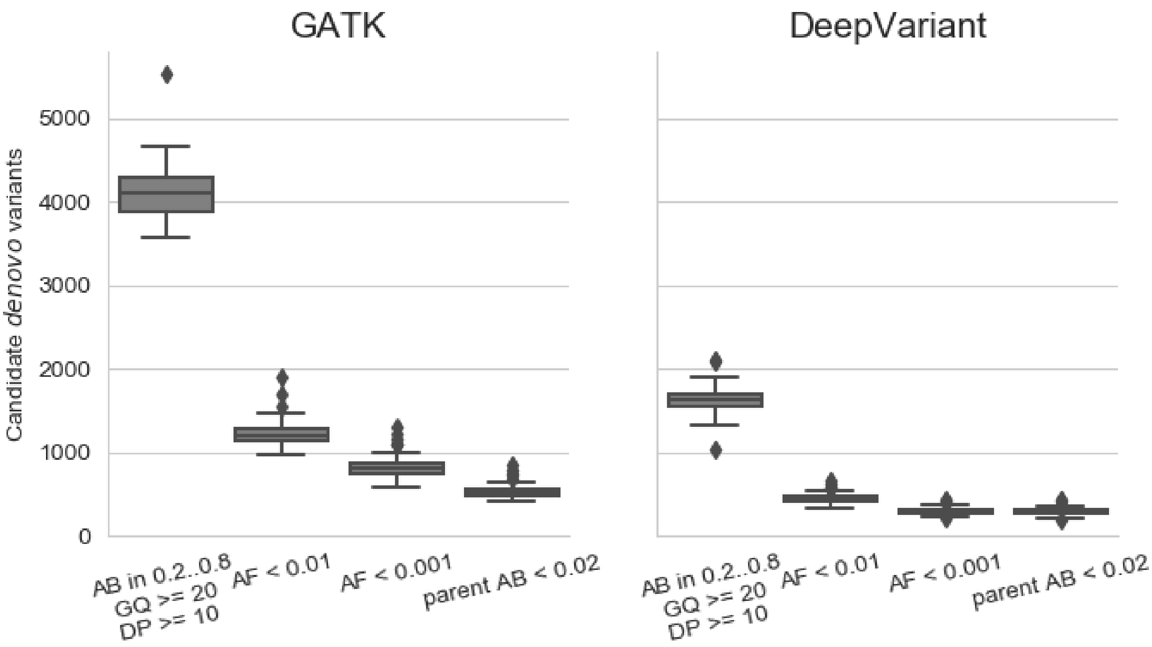

Supplemental Figure 13. Candidate autosomal de novo variants per genome identified by GATK and DeepVariant including low-complexity regions. Compare this to Figure 4 to see the effect on including or excluding LCRs.

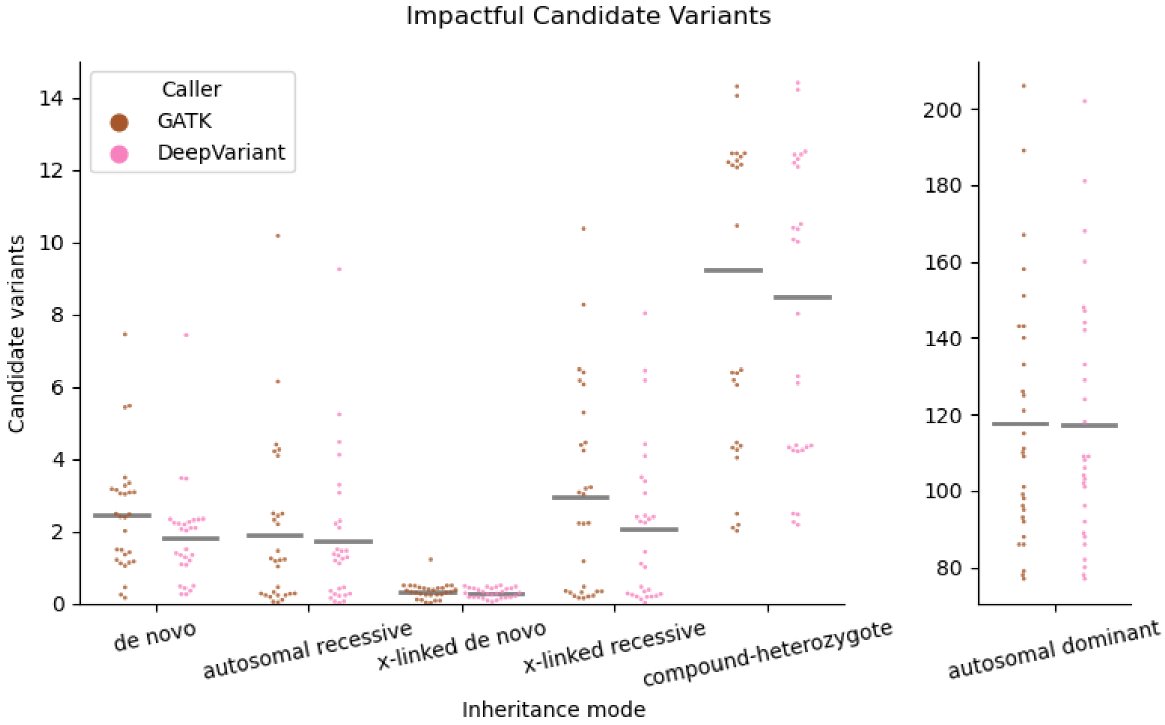

Supplemental Figure 14. Impactful variants in each inheritance mode for 49 sarcoma whole-genome trios. Only “impactful” variants as determined by slivar using annotations from

VEP, snpEff, or bcftools are shown. Counts for autosomal dominant variants are shown in a separate plot due to the much larger numbers. Each dot represents the number of candidate variants (y-axis) passing the inheritance mode (x-axis), genotype quality, population allele frequency, and allele balance filters for a single family. Gray bars indicate the mean number for each class and inheritance mode. We show this figure as validation of the data in Figure 5 in the manuscript.

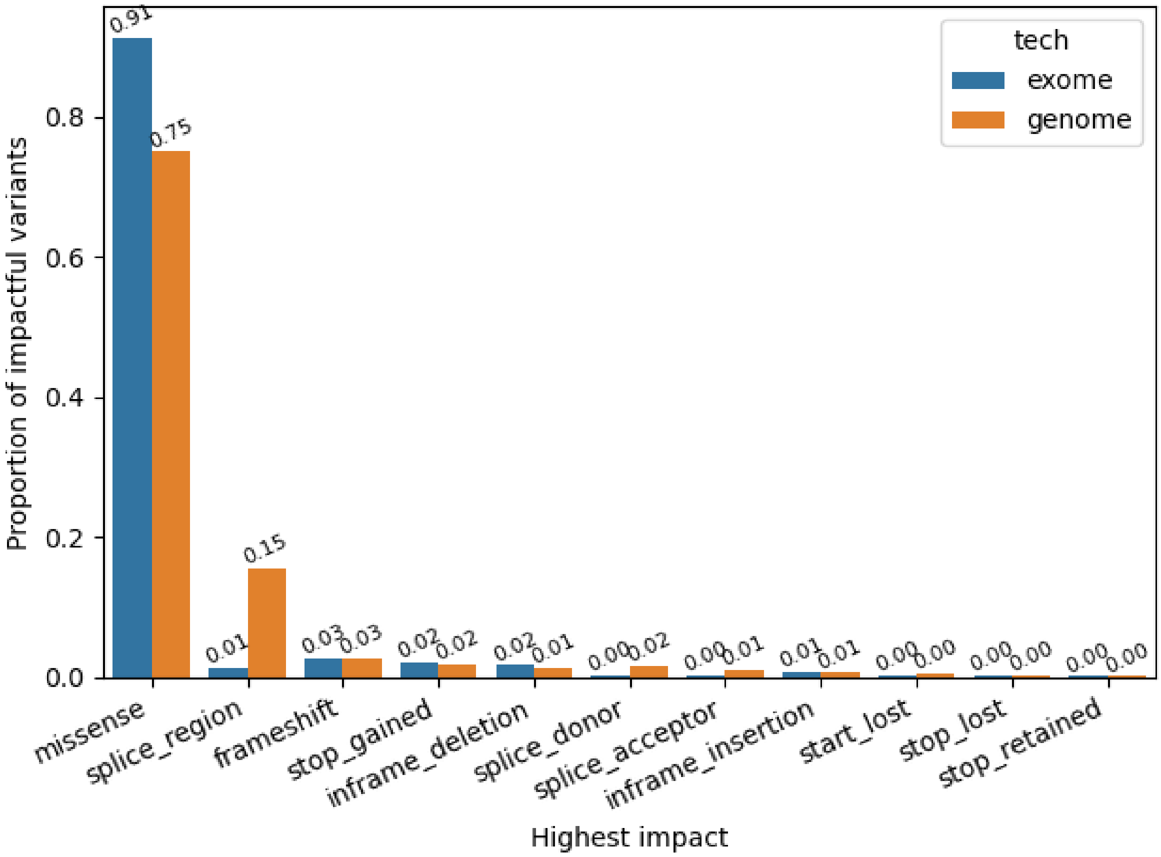

Supplementary Figure 15. Proportion of impactful variants in each class. Note that *genome* (in orange) has a much higher proportion of splice_region variants when compared to *exome* (in blue).

Supplementary Tables

Inheritance Mode	WES GATK	WGS GATK	WGS DeepVariant	WES GATK (impactful)	WGS GATK (impactful)	WGS DeepVariant	WGS GATK (genic except

						(impactful)	intronic)
de novo	1.4	200.3	152.9	1.1	2.2	1.6	12.8
Autosomal recessive	0.5	488.3	501.5	0.4	1.7	1.5	15.3
Compound heterozygote	7.3	129K	22.3	3.4	9.0	9.2	27.3
X-linked recessive	0.3	492.9	268.8	0.3	2.7	1.8	13.1
X-linked *de novo*	0.3	2.7	1.65	0.3	0.0	0.0	0.2
Autosomal dominant	102.0	18729.7	18505.9	68.9	117.8	117.4	738.8

Supplementary Table 1. Mean number of candidate variants for each inheritance mode for an exome and genome cohort. The exome cohort is 36 trios with CHD and the genome is a cohort of 49 trios with a sarcoma phenotype.

Supplementary Table 2. Impact order used by slivar. The sentinel values “IMPACTFUL_CUTOFF” and “GENIC_CUTOFF” separate impacts that are “impactful” and “genic”, respectively above each of those entries from those below.

transcript_ablation
splice_acceptor
splice_donor
stop_gained
exon_loss
frameshift
stop_lost
start_lost
transcript_amplification
gene_fusion
disruptive_inframe_insertion
disruptive_inframe_deletion
inframe_insertion
inframe_deletion
missense
protein_protein_contact
conservative_inframe_insertion
conservative_inframe_deletion
protein_altering
inframe_altering
splice_region
structural_interaction_variant
bidirectional_gene_fusion
incomplete_terminal_codon
start_retained
stop_retained
rare_amino_acid
5_prime_utr_truncation

3_prime_utr_truncation
`IMPACT_CUTOFF` is a special value that slivar uses to set INFO.impactful for any variant with
any CSQ that has an impact that falls above this sentinel
IMPACTFUL_CUTOFF
synonymous
gene
coding_sequence
mature_miRNA
5_prime_UTR_premature_start_codon_gain_variant
5_prime_UTR
3_prime_UTR
initiator_codon
miRNA
non_coding_transcript_exon
non_coding_exon
non_canonical_start_codon
nc_transcript
exon_region
conserved_intron
GENIC_CUTOFF
intron
NMD_transcript
non_coding_transcript
non_coding
upstream_gene
sequence_feature
downstream_gene
intragenic
TFBS_ablation
TFBS_amplification
TF_binding_site
regulatory_region_ablation
regulatory_region_amplification
feature_elongation
regulatory_region
conserved_intergenic
feature_truncation
intergenic
intergenic_region

Supplementary Discussion

Evaluation of Causal Variants in RGP

We evaluated variants deemed causal by the RGP consortium to evaluate which, if any, were removed by our recommended filters. All of the expected variants were recovered using our filtering parameters. There were however some variants that we expected not to recover, specifically CNV calls, cryptic inheritance patterns not evaluated here, or differences in the way alleles were reported in the VCF we analyzed. Specifically, there were 29 variants (or pairs of variants in the case of compound heterozygotes) that were identified as causal in the subset of RGP dataset analyzed in this study. Our filters recovered 23 of these candidate causal variants among the common inheritance patterns tested by slivar. However, the RGP manifest reported five copy-number variants leading to compound-heterozygotes that were missed, yet this is expected since the RGP VCF we used only included SNP/indel calls from GATK and DeepVariant. In those cases, however, our filters recovered the SNV/INDEL variant for each of the five compound heterozygotes. In addition, there was a RGP heterozygous variant for an oocyte maturation defect that was transmitted from an unaffected father to two affected girls; since this atypical scenario did not meet any of the tested inheritance patterns, it was not recovered by our search.
